# The work experience of male nursing teachers in Chinese universities: a phenomenological study

**DOI:** 10.3389/fpsyg.2023.1256934

**Published:** 2023-11-23

**Authors:** Zhenhua He, Jia Yao, Minerva B. De Ala, Xiaolan Zhang

**Affiliations:** ^1^Yuanpei College, Shaoxing University, Zhejiang, China; ^2^School of Nursing, Philippine Women's University, Manila, Philippines; ^3^Nursing Department, The First Affiliated Hospital of Zhejiang Chinese Medical University (Zhejiang Provincial Hospital of Chinese Medicine), Zhejiang, China

**Keywords:** male, faculty, nursing, occupational stress, job satisfaction, China

## Abstract

**Background:**

With the increase in the number of male nursing staff, more and more highly educated male nursing staff are joining the nursing higher education industry. Nevertheless, male nursing teachers in China are still a very small group in the education industry, but they have made important contributions to nursing education in China. Work experience is a key factor in the stability of the professional team and the quality of education. However, there is little research on the professional feelings of Chinese male nursing teachers.

**Objective:**

Explore the true feelings of male nursing teachers in Chinese universities about their work, understand their actual work difficulties, their sources of work stress and methods of coping with stress under the Chinese cultural background, and combine their professional expectations to provide ways to solve these problems, and help male nursing educators better apply nursing education work.

**Methods:**

This study uses an interpretive and exploratory qualitative research design method, in which the participants of male nursing teachers are selected from 30 universities with nursing majors in Zhejiang Province by the method of purpose sampling, and semi-structured in-depth interviews were used to collect data. A Van Manen process was used for data analysis. The 10th respondents were saturated.

**Results:**

Five themes were extracted in this study, namely realistic difficulties of career, positive feelings of career, sources of occupational stress, coping methods for occupational stress, and career development expectations.

**Conclusions:**

Male nursing teachers in universities can actively adjust their thoughts and behaviors and face negative professional feelings due to gender factors. But they still need more comprehension and support from educational institutions and society. The support of leaders and universities can help them better adjust to professional development and maintain a rational and stable nursing teacher team.

## Introduction

With the continuous deepening of China's medical system reform and the advancement of the construction of nursing disciplines, an increasing number of men have begun to engage in nursing work. According to the statistics of the “2018 China Health and Family Planning Statistical Yearbook” released by the National Health and Family Planning Commission, the proportion of registered male nursing practitioners in China rose from 1% in 2010 to 2.2% in 2017 (Shen et al., 2020). Men can be energetic, calm, and creative, and has other personality characteristics and practical advantages that are suitable for nursing. Men have become an indispensable part of nursing teams to meet the developmental needs of the nursing industry (Zhang and Liu, 2016).

At present, owing to the gradual increase in the number of men in nursing teams, they are not limited to clinical nursing work; an increasing number of men with high academic qualifications are engaged in the nursing education industry. However, because of the historical development characteristics of the nursing discipline, there were few male nurses in China before the 1990s (Zhang and Tu, 2020), and the service characteristics of the profession resulted in the feminization of nursing in society's inherent cognition (Liu, 2020). This perception has also directly affected the nursing education industry, and the current nursing faculties of Chinese universities are composed mainly of women (Sumpter et al., 2022). The presence of men in nursing education work is often questioned by the public; thus, male nursing faculty tend to show low levels of professional identity and high levels of burnout and willingness to leave, which is not conducive to the construction and optimization of the nursing education workforce. In recent years, some scholars have begun to realize the importance of male nursing teachers (MNT) and their development difficulties and have begun to increase their research investment in the development of male nursing teacher resources (Santos, 2020a). However, due to the small scale of MNT organizations and the recent development of such studies, current research on the development and management of male teacher resources in the nursing field is still insufficient (Santos, 2020b), resulting in a lack of understanding of male nursing teacher resources among education managers. There is still no effective basis for the development and utilization of such nursing education resources (Zeb et al., 2020), which is not conducive to the overall development and utilization of nursing education resources and hinders the construction and development of nursing teachers. Therefore, stabilizing resources and promoting increased numbers of MNT has become an urgent problem for college nursing education managers.

The construction of teaching staff is an important foundation for the professional development of nursing, and it is directly related to the quality of talent training and the level of development of the discipline (Kaasalainen et al., 2012; Collier, 2018). MNT is a specific group in nursing higher education. The particularity of group, profession, and gender can easily cause MNT to experience greater physical and mental pressure (Dos, 2020). The experience of MNT is different from that of female teachers. They face not only regular sources of professional stress, such as a heavy load of teaching tasks and scientific research, but also specific stressors, such as professional identity and gender bias. Against China's cultural background, which advocates that “silence is golden” for men (Bond, 1993), MNT's low professional identity, lack of social support, and professional environment and gender perspective differences lead to multiple problems, such as a “cognitive gap” (Collins and Stockton, 2018). Such issues indicate that we should pay attention to the professional experience of MNT, a special and important group, rather than simply combining them with female nursing teachers. However, no previous research has focused on the professional experience of MNT has been conducted.

Therefore, this study will use phenomenological research methods, from the perspective of MNT in Chinese universities, to restore the current professional status of MNT in Chinese universities, and to deeply discover the occupational stress, solutions, job satisfaction, career development expectations and faced by this group in a work environment dominated by women. Based on the real thoughts of MNT and the current development status of MNT, suggestions for the development of professional groups are put forward to provide reference opinions for educational managers and for MNT. Speak up to improve the professional environment for MNT.

## Methods

### Study design

Conceptual qualitative research (Collins and Stockton, 2018; Johnson et al., 2020) plays a vital role in exploring the nature of the MNT's professional experience. Phenomenology (Bliss, 2016; Kelly et al., 2016), as a qualitative method of experience research, emphasizes an open attitude toward investigating the roots of phenomena, seeking the connections between phenomena, and exploring the essence of experience. Practical experience emphasizes the problems of interpersonal interactions and relationships, making it especially suitable for phenomenological discussions. This study uses interpretative phenomenology (Suddick et al., 2020) to explore the professional experience of MNT in universities and the deep meaning underlying this phenomenon. It explores the experience of MNT in universities because it is the experience of MNT in the life world of nursing teaching organizations. It emphasizes interpersonal interactions and relationships, making the use of phenomenology suitable for discussion. Interpretative phenomenology recognizes that existence is affected by situations and advocates understanding phenomena through explanations (Guillen, 2019). Work experience usually occurs in the workplace and in interpersonal interactions. Therefore, work experience is affected by various factors, such as the working environment, interpersonal relationships, and organizational atmosphere. In addition, work experience is closely related to aspects of the external environment, such as cultural concepts and social roles (Sue et al., 2009). Therefore, in this research, hermeneutical phenomenology is suitable for exploring the relationship and interdependent meaning between situations and professional experience.

### Ethical considerations

All materials in this study are stored in a special computer, and the computer password and file password are set to double switches, and only the researcher can open the computer and the file. When the research is completed, the researchers will immediately destroy the research-related materials to protect the privacy of the participants. In addition, the participants in this study fully understood the research background, purpose, methods and other information before participating in the study, and signed the informed consent form. This study strictly complied with the requirements of the Declaration of Helsinki and was approved by the Ethics Committee of the Affiliated Central Hospital of Shaoxing University.

### Participants

This study was carried out from July to December 2022 and used the purposive sampling method (Benoot et al., 2016) to select MNT from universities in Zhejiang Province as the research object. The participants in this study who met the inclusion criteria were MNT who had been engaged in university nursing education for 1 year or more. They needed to have a university teacher qualification certificate. The participants who met the inclusion criteria were recruited mainly through referrals by their peers. First, the researcher introduced the research purpose to the nursing teachers' peers (the nursing peers, as the referrers had no gender restriction) and identified those who met the inclusion criteria of being MNT. The nursing colleagues then provided a preliminary introduction to potential participants in this study. After a participant consented, the researcher introduced the purpose, content, and methods of the research in detail through the WeChat platform. The participants were asked to decide whether to participate in the study. If they participated, they had the right to withdraw at any time with no impact on or consequences for them. We read and explained the relevant content of the informed consent form to the participants, and the participants signed the form and voluntarily returned it to us. The sample size was determined by the criterion that the participants' information was repeated so that no new themes were presented in the analysis of the information (Sutton and Austin, 2015), and no new information emerged after we reached the 10th respondent in the interview; thus, a total of 10 participants who met the inclusion criteria were included in this study.

### Data collection

For data collection, we used a face-to-face semi-structured interview method (Jamshed, 2014). Based on the research questions and research purposes of this study, a preliminary interview outline was drawn up after consulting the literature and after repeated discussions and revisions with three qualitative research experts. In this study, two pre-interviews were conducted before formal interviews. After the pre-interview, the researcher invited the pre-interview subjects to express personal opinions and suggestions on the spot and listened to the recording repeatedly to reflect on the deficiencies in the interview process. Through the pre-interview, the researcher improved and adjusted the interview outline and interview methods. In the follow-up formal interview process, the interview outline was revised and improved according to the problems that appeared. The interview outline for this research included the following: (a) Can you talk about why you chose to engage in nursing education? (b) What do you think about nursing teaching in universities? (c) What impact do you think male identity has had on your nursing teaching work? (d) How did you deal with these influences? (e) How do you hope that men will develop better in nursing teaching?

The interview time was determined according to the participants' wishes based on the time they could arrange to be free and energetic. The distribution of interview time periods was five cases in the morning, three cases in the afternoon, and two cases in the evening. The interview locations were quiet and comfortable, relatively private interview rooms, cafes, and other places chosen mainly for the convenience of the participants. The interviews comprised four cases in Hangzhou City, two cases in Wenzhou City, two cases in Shaoxing City, one case in Quzhou City, and one case in Huzhou City. The specific interview locations included five cases in a conference room, two cases in an office, two cases in a café, and one case in a classroom.

For the interview, we prepared the following necessary materials and tools in advance: informed consent form, recording device, pen, and notebook. We arrived at the interview site 15–30 min in advance of the agreed-upon time, found a quiet and private seat, and reread the interview outline. Before the interview, we explained the purpose and significance of the research and the research process to each participant and asked them to sign an informed consent form after obtaining consent and again explaining the recording requirements. We recorded the entire interview after obtaining the consent form. At the beginning of the interview, the hot spot was determined through content other than the subject of the interview. Interview techniques such as repetition, clarification, and inquiry were used reasonably, and key content and non-verbal information were recorded to enrich the interview details (DeJonckheere and Vaughn, 2019). At the end of the interview, the participants were asked if they needed to add content, and we indicated that there might be a second interview and subsequent assistance in verifying the research content. In this study, 10 MNT participants were interviewed. Each interview lasted approximately 35–60 min, and all interviews totalled approximately 408 min, with an average of 40.8 min per case.

### Data analysis

After the interview, the researcher transcribed the interview recordings verbatim into textual materials. All interview recordings were transcribed within 24 h. During the transcription process, the researcher repeatedly listened to the recorded data, marked non-verbal information such as tone, pitch, and paused thinking, and checked the interview notes to ensure that the information was correct. The transcribed text was processed for privacy, and document formatting, such as page numbers and other information, was added. In addition, the researcher entered the general information of the participants and reflective diaries into the Word software.

Data collection and data analysis were carried out simultaneously; that is, the data were analyzed after completing an interview. This study used the Van Manen data analysis method (Heinonen, 2015). In the first step, at the beginning of the data analysis, after repeated reading of the text materials transcribed after the first three interviews, the interview notes and interview notes were combined to gain a sense of overall intuition. In the second step, we chose to encode meaningful statements in response to the research question, focusing on grasping the inner experience of MNT in their daily work and extracting meaningful units, see Table 1 for coding examples. The third step was to fully understand the connotation of each meaning unit, group similar or same-question codes together, immerse myself in the interview data for repeated comparisons, reduce repetitive or irrelevant meaning units, and summarize similar meaning units. The fourth step was to summarize and sort out the various themes and subthemes based on a summary of the previous meaning units and the clustering of the subject groups. The researcher strengthened the rationality and objectiveness of the subject analysis process through discussions with team members and comments from non-peer scholars, an example of thematic analysis is shown in Table 2. Finally, themes and subthemes were formed. In the fifth step, the initially obtained themes and subthemes were again checked against the textual material and units of meaning to determine whether the themes reflected the essence of the phenomenon, to continuously analyze the internal logic and relationships between the themes, to establish the themes and find corresponding examples of extracts from the material, and to form an explanatory text. The final step was to return the obtained themes and subthemes to the participants for verification and asking them to confirm whether these results accurately reflected the true feelings of the male nursing teacher about his work.

**Table 1:** Coding example.

**Participant**	**Related statements**	**Original encoding**
Participant 2	I only have more than 5,000 yuan a month, which is enough to pay off the mortgage every month. I hardly spend any money, I ate at school and didn't buy clothes. Other family expenses depend on my wife's salary. I was very disappointed with my job and felt really helpless, but I didn't dare to resign easily.	The salary is only 5,000 yuan Salary is not enough to pay off the mortgage Family consumption depends on wife's salary Feeling of career loss due to low wages Don't dare to resign
Participant 3	There was only one male nursing teacher at our school. I encountered many female teachers in the teaching and research section, who asked for leave during pregnancy. I am embarrassed to refuse... I need to undertake the teaching tasks of four professional courses in one semester. Physical and mental exhaustion (oh)	There are very few male nursing teachers in schools Many female teachers take leave due to pregnancy Lack of teachers leads to heavy class load High physical and mental stress
Participant 4	I was sick and asked to leave my illness. Another male teacher has taken on many courses, and the female teachers have heavy teaching and family burdens, so the courses I am in charge of can only be temporarily suspended, waiting for me. After I recover, I will arrange my own time to make up for classes. No one will help me. I am actually very disappointed. I can only take care of all things on my own.	It's difficult to take time off when he's sick Male teachers have a lot of class hours, while female teachers have more families If you ask for sick leave, you can only suspend classes Disappointment at not being able to get help from colleagues

**Table 2:** Examples of thematic analysis.

**Summarize similar units of meaning**	**Refining theme groups**	**Subtheme**	**Theme**
There are very few male nursing teachers in schools The school's first male nursing teacher There was no male nursing teacher before me The office is full of female teachers Easily isolated by female teachers	Few teachers	Imbalance of occupational gender ratio	Realistic difficulties of career
Lots of class hours It's hard to take time off when you're sick Help other teachers with classes Taking into account both theoretical and experimental teaching Four courses in one semester Many female teachers take leave due to pregnancy The workload exceeds the acceptable range	Heavy workload		
My salary is very low The salary is only 5,000 yuan My salary is not enough to pay off the mortgage Great financial pressure Salary can only meet daily consumption Need financial assistance from parents	Low salary income	Contradiction between wages and gender roles	
I am the breadwinner of the family I am father and son Men are the main breadwinners of the family Family consumption depends on wife's salary Need to spend money from parents	High family financial pressure		

### Self reflection

Qualitative research uses the researcher himself as the research tool. The researcher's role and cognition have an impact on the research results. Therefore, the researcher has reflected on his role. I am a nursing teacher, male, married, with experience in social work. During my previous work practice, I have had close contact and in-depth communication with male nursing teachers. I have also personally experienced organizational silence in the workplace, so I play the role of an “insider” in this study. However, since I did not read any relevant literature on male nursing teachers in mainland China before doing the research, I did not have a complete understanding of the current working situation of male nursing teachers. Therefore, on a cognitive level, I may be an “outsider” to the study of the current working conditions of MNT.

In order to prevent my personal cognition from affecting the research results, before this study was officially launched, I recorded my existing views on the current work status of MNT, and compared them when I encountered similar codes during the data analysis process and examine, carefully understand and analyze respondent data to reduce the impact on research results. After this study was officially launched, I avoided reading literature related to this study to avoid being contaminated by the results of other studies. During the interview process, I will try to avoid expressing personal opinions or talking to male nursing teachers to avoid being induced by male nursing teachers. As the research gradually deepened, my understanding of the research issues was re-rooted in the qualitative data collected, and some personal opinions were gradually diluted or even replaced. Therefore, researchers gradually become objective and open-minded in their analysis and understanding of qualitative data, ensuring the scientific nature of research results.

In this ambivalent identity may have a subjective impact on the meaning of the experience. Therefore, researchers should constantly conduct self-reflection during the research process and record their own thoughts and ideas, that is, write reflective notes during the research process. Through self-reflection and recording personal prejudices, they can open their minds and give up their preconceptions before data analysis. In the process of data analysis, when I encounter an understanding that particularly matches my expectations, I will review my reflection notes, repeatedly compare and confirm whether my understanding and interpretation of the data are objective and correct, thereby minimizing my own beliefs impact on the results of this study.

## Results

A total of 10 participants who met the inclusion criteria were included in this study. Their basic information was as follows: age: 28–43 (34.80 ± 4.53) years; working time: 1–19 (8.40 ± 5.87) years; engagement in college nursing educational working hours: 1–19 (8.20 ± 5.86) years; annual average number of teaching hours: 190–510 (365 ± 113.51), participant characteristics is shown in Table 3.

**Table 3:** Participant characteristics.

**Participanta**	**Age (years)**	**Education**	**Marital status**	**Working years (years)**	**Nursing education time (years)**	**Title**	**Teaching hours per year (hours)**
Participant 1	28	Master	Unmarried	1	1	Assistant	230
Participant 2	32	Doctor	Unmarried	3	3	Instructor	470
Participant 3	34	Doctor	Married	5	5	Instructor	420
Participant 4	39	Master	Married	12	11	Associate Professor	310
Participant 5	34	Master	Married	9	9	Instructor	490
Participant 6	29	Master	Unmarried	2	2	Assistant	260
Participant 7	40	Doctor	Married	17	17	Associate Professor	300
Participant 8	43	Doctor	Married	19	19	Associate Professor	190
Participant 9	36	Master	Married	10	10	Instructor	470
Participant 10	33	Master	Married	6	5	Instructor	510

**Figure 1:**
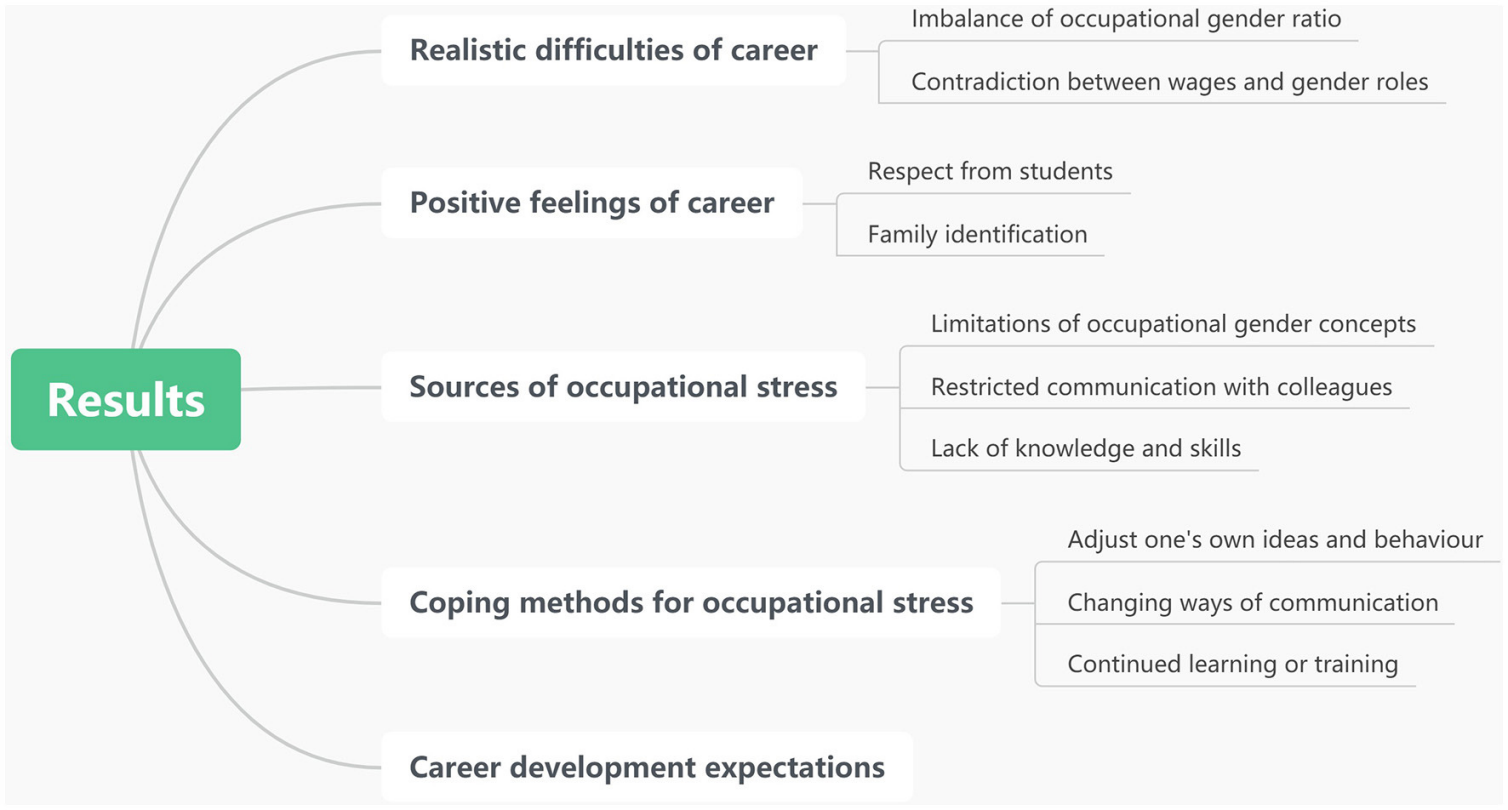
Extracted subthemes and themes from data.

Through inductive analysis of the research data, data analysis revealed five main themes and 10 subthemes. The specific descriptions are shown in Figure 1.

### Theme 1: realistic difficulties of career

The participants shared various difficulties encountered in daily teaching owing to gender factors. They also stated a contradiction between work and family gender roles. These practical difficulties were all related to participants' negative feelings about their profession. Most participants complained that the teaching workload was large, but the income was low.

#### Subtheme 1: imbalance of occupational gender ratio

The imbalance of the occupational sex ratio refers to the occupational pressure and instability caused by the excessive workload associated with the lack of male teachers in the nursing teacher group. The participants said that the nursing staff was mainly female, there was a shortage of MNT, and the lack of a reasonable occupational gender ratio had caused MNT to bear heavier teaching burdens owing to teacher shortages. The accumulation of workload pressure can easily induce negative inner career experience.

*There was only one male nursing teacher at our school. I encountered many female teachers in the teaching and research section, who asked for leave during pregnancy. I am embarrassed to refuse... I need to undertake the teaching tasks of four professional courses in one semester. Physical and mental exhaustion (oh) (Participant 3)*

*I am in charge of two theoretical courses and three nursing practice courses. This is horrible. What can be done about this? I do not want to..., in fact, I think very tired (Participant 6)*.

Excessive teaching workload has always been the main source of negative work experience among nursing teachers. Under the traditional Chinese concept that “men should bear more' the workload of MNT often exceeds their own capacity. However, under the image of a male role of “bearing hardships and standing hard work,” most Chinese MNT will choose to bear the high pressure of work silently, which physically and mentally exhausts them during work.

In addition, the participants talked about their inability to obtain support and help from colleagues in their teaching work when they were ill. In a working environment with a shortage of teachers and a low ratio of male teachers, most MNT are overloaded, and female teachers cannot provide work assistance due to family factors.

*I was sick and asked to leave my illness. Another male teacher has taken on many courses, and the female teachers have heavy teaching and family burdens, so the courses I am in charge of can only be temporarily suspended, waiting for me. After I recover, I will arrange my own time to make up for classes. No one will help me. I am actually very disappointed. I can only take care of all things on my own. (Participant 4)*

Illness often places individuals in a physically and psychologically vulnerable state. MNT choose to suspend classes due to illness and rarely receive help from their colleagues. This will reduce their professional sense of belonging, and “silence is golden” against the cultural background of China. MNT tends to accumulate this feeling internally, and the accumulation of long-term negative experiences can lead to professional exhaustion.

#### Subtheme 2: contradiction between wages and gender roles

Occupational income is the main source of funds to maintain the family's daily life, including daily expenses such as food, transportation, housing, raising children, and supporting parents. In Chinese society, male income is usually the main source of life for the family, but the current income of ordinary university teachers is low. The respondents generally reported that there is a real contradiction between salary income and gender roles in the family, and their salary income cannot meet their daily family expenses, increasing their sense of powerlessness in life. Most of the participants in this study regarded low income as their greatest difficulty in the work process. They were unable to cover the daily expenses of their families with their current occupational income and needed financial assistance from other family members, such as wives and parents. In addition, in the context of high housing prices, housing loans or saving money to buy a house have also become primary sources of pressure on expenditures.

*I only have more than 5,000 yuan a month, which is enough to pay off the mortgage every month. I hardly spend any money..., other family expenses depend on my wife's salary...very disappointed, really helpless, but... (Participant 2)*

*As a son, husband, and father, I should..., need to bear greater economic pressure in life, but my salary is very low...; my salary is far from enough for our family daily expenses. My wife's..., my parents' retirement salary is also spent in our house, I feel... (Participant 3)*

*I have been working for 10 years, my college classmates..., but my salary can only support daily expenses. I still live in the school dormitory with my wife and children. In order to buy a house, I have to apply for more courses, instruct students in competitions and scientific research declarations... Is there any way to save money? (loss expression) (Participant 9)*

The contradiction between occupational income and daily expenditure has an important impact on professional experience. As the cost of social life increases, the contradiction between the two becomes increasingly prominent. MNT are usually the main income earners of the family; thus, they experience financial constraints. Therefore, they need to adjust their expenditure structure and income model in time and do a good job of personal psychological adjustment regarding family roles to achieve a good balance between life and work.

### Theme 2: positive feelings of career

Professional positive feelings refer to the psychological pleasure produced by the MNT's professional recognition by society. Students and their families are the main social relationship group and support system for MNT, and their recognition is also an important source of positive professional feelings toward MNT. The participants shared that they received recognition and respect from students and formed good teacher-student relationships. Additionally, their careers were affirmed and supported by family members. The recognition of these social relationships helped the MNT form a positive professional experience.

#### Subtheme 1: respect from students

The attitudes and evaluations of students are manifestations of the comprehensive ability and personal charm of the MNT. The participants said that they received various forms of respect and recognition from students, which positively guided their professional psychological feelings. The participants said that they received good teaching evaluations and even holiday blessings from students.

*The students were very considerate of the work of our male teachers. They..., my final teaching evaluation is often better than that of the female teachers....I am very happy... and often receive blessing text messages and cards from students. (Participant 7)*

The participants felt that the students were serious, studious, and respecting teachers in the classroom, and harmonious relationships between teachers and students brought great professional satisfaction to nursing teaching work.

*The students are serious in class and respect our male nursing teachers very much. (Participant 8)*

The performance of respect in the classroom may be due to the role gap between the teacher and the student and the teacher's authority, and the good interaction between the teacher and the student after class is the true performance of the student's respectful behavior. The participants said that students would take the initiative to greet them outside the classroom, showing that they respected and recognized their teachers.

*Nursing students greet me enthusiastically when they meet me on campus or on the street. They think... it will not be different because I am the only male teacher, I think.... (Participant 10)*

Students are the main element of the teaching relationship, and respect from students is a full affirmation of this relationship. Student respect brings recognition of their profession to MNT, and this recognition is also an important component of positive professional feelings.

#### Subtheme 2: family identification

Family relationships are the main component of the social support system of the MNT, and the family's attitude toward their occupation is also an important factor that affects their professional experience. The participants said that their families had a high degree of recognition of the profession of nursing teachers and encouraged them to work hard. The recognition of family members was also an important motivation for work.

*My parents think that it is a good career for boys to engage in nursing education in colleges and universities...proud.... I must work hard and contribute my strength to my posts. In the future... (Participant 5)*

The participants also mentioned that their wives played an important role in their career development. Wives' contribution to the family can reduce their worries and worries so that they can gain more experience in putting into work.

*My wife is very supportive of my work. My wife... has made many contributions to the family. She took good care for the elderly and children in her family. She often tells me... This also makes me less worried so that I can concentrate on teaching. (Participant 9)*

The influence of family members on their work is important. In the process of professional development, it is beneficial to obtain recognition and support from family members. Family members with a full understanding of professional values and being willing to provide support and help in life can affect MNT's attitudes and commitment to the profession because some people will fail in their jobs because of trivial family matters, producing long-term and lasting negative work experiences.

### Theme 3: sources of occupational stress

Occupational stress refers to the stress factors that MNT suffers from different channels in the work process, which causes them to express professional stress in spirit or behavior, such as resistance or boredom. Male teachers belong to a minority group in nursing teaching organizations, and their teaching objects are composed mainly of women, which also causes gender factors to have a certain impact on their professions. The participants shared the limited cognition of parents and other professional teachers regarding the gender concept of the nursing profession, noting that it even caused gender gaps in communication. Some participants believed that they had insufficient abilities and gender disadvantages in nursing teaching.

#### Subtheme 1: limitations of occupational gender concepts

Most nursing teachers in universities are women, and men are a minority group. The general public has a certain gender bias against MNT because of the inherent cognition of the feminization of nursing in society. The participants said that they had been subjected to occupational gender prejudice by their parents and other professional teachers, which made them feel loss and embarrassment about their gender at work.

*At teacher meetings, I can always hear other professional teachers talking; they say...there are male teachers in the nursing profession,...very strange...and still a doctor, in my heart...uncomfortable.... (Participant 2)*

*...The parents of students said how a boy could come to be a teacher in nursing, which made me feel very embarrassed. I feel very disappointed. (Participant 3)*

Society's prejudice against MNT's gender is also a true manifestation of the current professional development status of this group, and this limitation of social cognition will interfere with the professional psychology of MNT, causing them to experience psychological stress in relation to their gender. In addition, resistance leads to occupational stress.

#### Subtheme 2: restricted communication with colleagues

The interpersonal relationship between colleagues is an important factor that affects professional experience. Gender differences can easily cause psychological and behavioral distance, while long-term estrangement can easily cause occupational anxiety and loneliness. The participants said that they could not integrate into female teachers' organizations due to gender factors, and they felt lonely in interpersonal relationships.

*I usually act alone in meetings or team-building activities in the teaching and research sections. I also... cannot integrate well into the female teacher team and often feel lonely. (Participant 6)*

Some female teachers maintain psychological distance and behavioral resistance to MNT because of their personality or gender cognition. Long-term distancing can easily lead to gaps and even contradictions between colleagues.

*Some female teachers have a strange look at me,...ignore me..., and their attitude toward me is not very friendly. Once..., I felt very deep in my heart. Repulsion and anxiety affect my work mood, and I sometimes feel anger. (Participant 10)*

Interpersonal estrangement among colleagues is a common feeling among MNT, and this long-lasting and depressed feeling will continue to impact their professional psychology and attitude. Even if a harmonious interpersonal relationship is maintained on the surface, once this potential stressor emerges, it is difficult to control. Therefore, improving the relationships between colleagues is also very important.

#### Subtheme 3: lack of knowledge and skills

Nursing is a subject with highly specialized knowledge and skills, which requires nursing teachers to have a wealth of professional knowledge and skills. Interpersonal communication is an important skill in nursing practice. Under the influence of the “silence is golden” cultural background, men are usually not good at speaking, which also leads to shortcomings in teaching skills.

*Nursing is a subject that requires humanistic care and communication. As a male, he is not good at speaking and cannot teach students and patients communication skills and abilities well. We hope to receive guidance and help from senior teachers. (Participant 2)*

In addition, most basic nursing skills are refined operations, and MNT has certain disadvantages compared with female teachers because of the characteristics of crude gender behavior. However, this disadvantage can be compensated for by long-term training.

*Nursing involves many operations. There is a gap between the fine operations, such as bed making, infusion, intramuscular injection, and female teachers, and there are other... I often practice during breaks, and I believe that through hard work... (Participant 3)*

Professional nursing knowledge and skills are the basis for maintaining professional behavior. When this foundation is missing, its absence will lead to backwardness in nursing teaching behavior, and backward behavior will affect psychology and add stressors. Therefore, learning and mastering professional nursing abilities are very important in the careers of MNT.

### Theme 4: coping methods for occupational stress

In the face of stress, the human body produces instinctive coping behaviors, and individual coping behaviors vary greatly depending on personal characteristics, living environment, and experience. Effective coping methods can properly deal with the impact of work pressure, thereby establishing a positive work experience. The participants shared that in the face of work pressure, they had to adjust their own role concepts in a timely manner, change their behavior, and face doubts and prejudices with a positive attitude. Some participants also believed that continuing education and learning is an important way to improve their abilities to better adapt to work and reduce the pressure caused by ability gaps.

#### Subtheme 1: adjust one's own ideas and behaviors

A work stressor is a dynamic change that requires an efficient response method. An individual's self-concept guides their behavior. In the face of work pressure, an individual can adjust his or her own concept over time, establish a positive mental state, and resolve the source of stress by changing his or her behavioral pattern to achieve an effective behavioral ending. The MNT must recognize the value of their profession internally. The development of the nursing profession requires the participation of all nursing staff, and this kind of participation is not sex-specific.

*Learn to adjust my own professional concept; I often tell myself that the nursing discipline needs men to join in order to achieve more comprehensive development. (Participant 3)*

When facing doubts, we must take the initiative to allow others to understand us, show our own values and advantages, respond to others' questions and doubts, and gain support and trust from the outside world.

*When facing the parents of students, I often... also take the initiative to introduce myself and let everyone know me and show myself...and to the parents of students, I am also very supportive and believe in my ability to work.... (Participant 5)*

The outside prejudice against MNT is real, and it is difficult to actively make it disappear or resolve it. It is necessary to adapt to this pressure individually, and self-adaptation must usually be achieved by adjusting individual concepts and behaviors. Therefore, the MNT should establish a correct view of occupational gender and face pressure with a positive attitude and behavior to realize the relief of pressure.

#### Subtheme 2: changing ways of communication

Communication style is an important behavioral manifestation of interpersonal relationships. A positive communication style can shorten the distance between people, eliminate interpersonal barriers caused by gender differences, form harmonious interpersonal relationships in the workplace, and stimulate positive professional feelings. Active interpersonal communication can improve the relationship between male and female teachers.

*I took the initiative to change. I took the initiative to greet female teachers every day when I went to get off work so I could get along with them better than before. (Participant 2)*

In addition, actively sending friendly behavioral signals can change others' interpersonal attitudes. Mutual help and cooperation can enhance interpersonal assistance relationships, strengthen others' recognition, and form a good workplace relationship environment.

*I took the initiative to adjust classes or undertake teaching tasks when the female teacher was pregnant or when the child asked for leave. There was a semester... the female teacher thought that the existence of a male teacher was also necessary. (Participant 6)*

*I actively asked female teachers to undertake teaching tasks and scientific research activities together to strengthen their understanding and recognition of me in the work. I also learned technology. (Participant 10)*

Changing the way of communication can strengthen cooperation and learning between teacher groups, improve interpersonal relationships between nursing teachers of different genders, form a good professional environment, develop positive professional feelings, and stabilize the teacher group.

#### Subtheme 3: continued learning or training

Continued learning or training is an important way for an individual to improve his/her own abilities and is also an effective way to promote professional development. The improvement of one's own abilities can meet professional needs, reduce sources of professional pressure, and lay a good foundation for future development. The practice of basic nursing skills can compensate for shortcomings in an individual's own abilities, and the strengthening of superior skills can give them an advantage.

*I need to continue working hard. On the one hand, I will strengthen the basic nursing practice to narrow the gap with female teachers. On the other hand, I will strengthen male-dominated operation items, such as operating room nursing. I think... helpful. (Participant 3)*

The nursing discipline has strong clinical characteristics, and regular clinical practice helps maintain advanced and clinical nursing skills and prevent poor nursing teaching and clinical practice.

*My main research direction is wound care, which has greater advantages for men, so I will regularly go to the wound care clinic of the affiliated hospital to learn to improve my clinical skills and nurse-patient communication skills. (Participant 5)*

Most nursing teachers in China currently have a low educational background. The promotion of MNT through doctoral studies is conducive to the formation of academic advantages and plays an important role in ability improvement and career development.

*I am participating in an on-the-job doctoral study According to my understanding, male nursing doctors are relatively rare in China. I hope to gain certain advantages for future scientific research and promotion. A few years later... associate professor.... (Participant 10)*

Continued learning or training can improve the comprehensive ability of MNT, enable them to meet the professional ability requirements of nursing teachers, make them more versatile at work, and create career development advantages. Therefore, MNT should continuously build their own capacity, make up for their shortcomings, give play to their gender advantages, and promote the advancement of the nursing discipline.

### Theme 5: career development expectations

Career development expectations refer to the future needs of MNT based on their actual feelings about work and are usually the most strongly desired goals. Achieving these expectations requires the cooperation of external parties to be effectively achieved. The participants shared that a perfect occupational gender ratio is beneficial to the development of teaching work and allows them to experience a sense of belonging to the group.

*I hope that more male nursing teachers can be introduced, and the ratio of nursing teaching and research sections can be improved so that we can better carry out our work and give us a certain sense of group membership. (Participant 4)*

At present, the majority of nursing teachers in Chinese universities have master's degrees. Therefore, a Ph.D. is also the goal of many nursing teachers. They can improve their academic qualifications and abilities through doctoral studies to lay a foundation for future career development.

*I also want to go to my PhD when I have the opportunity. On the one hand, I want to improve my academic qualifications, but more importantly, I want to improve my abilities. This is...helpful. (Participant 6)*

The meager salary makes it difficult for MNT to carry the burden of their family role, and it is the problem that they are most eager to solve at present.

*Our salary is too low; we are working...no house...I also hope that the school leaders can consider our current situation of life. (Participant 10)*

Career development expectations are very important, which also reflects the fact that MNT has requirements and goals for the profession they are engaged in. This finding should enable education managers to better grasp the psychological needs of MNT, formulate reasonable career development plans and management plans for this group, and promote the stable and harmonious development of the nursing teacher team.

## Discussion

This study found that recognition of one's own profession is the original motivation for MNT to choose this job, including their love for the nursing profession and their inner positive professional attitude. As Manen (2014) said, phenomenology is the study of the meaning of life experience, and all meanings of research originate from the cognition of life experience. Employment experience is an individual experience constructed during the employment process. In the field of education, Manen and Adams (2010) believes that research is a phenomenological and educational enterprise that has nothing to do with life, but a way of looking at life. MNT put forward their true inner feelings through their long-term feelings about being engaged in the nursing education profession, which is a way of looking at their work. During the interviews, most participants expressed that they faced social stigma and social prejudice due to gender roles in the nursing industry, as well as pressures such as low wages, heavy workload, insufficient professional capabilities, low academic qualifications, difficulty in professional title promotion, and low social support, so they had a negative impact on their nursing education career, but they still show a positive attitude toward the nursing education profession, are satisfied with the relationships between teachers, students and colleagues, and are full of hope and hope for the future development of nursing education. This also echoes the research results of Santos (2020a), MNT have a strong sense of professional belonging, support from parents and colleagues, and social experience, which can enable them to overcome gender discrimination at work and face it with a more positive attitude. Collie (2021) research results show that male teachers in universities face greater occupational pressure than women, and the utilization rate of social support is also low. The social support that university teachers receive, especially the care among colleagues, is directly proportional to their teamwork ability (Krammer et al., 2018; Collie, 2021).

Manen (1982a) proposes in Phenomenological Pedagogy that as a result of theoretical overlap and perspective, the truth remains hidden and we tend to look for principles once theoretical schemes are put into practice. Analyzing the reasons from the hidden truth, as society gradually strengthens its recognition of male nursing roles, the increase in male nursing workers can stimulate their group consciousness, find their own professional belonging, and improve professional satisfaction (Arif and Khokhar, 2017). Manen (1982b) inspiring theory mentioned that in order to serve the good, many human behaviors are performed for the inner mission, and they follow their inner beliefs to implement good behaviors. The sense of mission and spiritual beliefs of MNT have a significant positive impact on nursing educators (Gravens and Goldfarb, 2020). It is precisely because of their inner love that they choose to engage in the profession of nursing teachers, allowing them to stick to their jobs, even if they encounter difficulties at work. Despite many difficulties and even gender discrimination, MNT can stick to their inner beliefs and fight for the cause of nursing education. Teachers' professional experience directly affects their teaching quality and scientific research results, and has an important impact on their career stability and loyalty (Toropova et al., 2021).

According to Manen (1990) Beyond Assumptions, only when practice is solved can theory make room for itself and put theoretical research into practice. Therefore, this study provides targeted practical policy suggestions for the development of male nursing teachers based on the interview content and the current situation of nursing education management in China. It is recommended that school administrators strengthen organizational support for MNT, such as encouraging the expression of MNT's emotions, conducting regular psychological surveys and health check-ups, understanding their true professional experience and development expectations, and actively conveying to them that schools and society care about MNT, a special group of university teachers. Additionally, they should promote the organizational integration of different teacher gender groups through teaching and research cooperation, team building, and other efforts to promote the physical and mental health of MNT.

In the face of the current deployment of nursing teachers in universities, changing the professional gender perspective and ratio is a problem that school administrators urgently need to study and solve (Solbrække et al., 2013). On the one hand, schools need to strengthen internal and external publicity about nursing majors, focus on improving the awareness and social positioning of nursing majors, and strengthen the appeal of nursing to men through activities such as public elective courses for MNT and lectures by male nurses in the community. On the other hand, schools should actively mobilize enthusiasm and participate in the MNT. Research has shown that improving the gender ratio of teachers can effectively improve the quality of subject development and the stability of professional teams (Dowling, 2006). Managers should increase the number of MNT and formulate relevant policies, introduce and train MNT in a targeted manner, and increase their number, education level, and proportion of professional titles so that they can truly feel that they are important in the development of nursing education. This approach will enhance the professional identity and sense of benefits of MNT.

In view of the differentiated needs of university nursing teachers of different genders and levels, a standardized teacher training program and promotion system have been established to provide an individualized career development platform. Younger teachers are coordinated and trained at the school level, training programs and assessment plans are formulated, and targeted training is carried out by senior teachers through instruction, collective lesson preparation, and business learning. To address the problem of imbalanced professional title matching, clinical teachers can be invited to take part-time classes in school (Bifftu et al., 2018) to focus on cultivating and strengthening students' clinical skills, case analysis, and nursing innovation and enhancing professional application capacity building. At the same time, we should increase policy support, implement a program to encourage young teachers to pursue doctoral degrees, and provide information about excellent doctoral degree programs at other schools to improve the overall low level of education of teachers.

## Conclusion

This study focuses on the MNT's feelings about their profession. Teachers are an important foundation for the development of nursing education. Doing research on the development of teacher groups is of great significance to promoting the improvement of the quality of nursing education. Since male Chinese account for a small proportion of the nursing educator group, research on the career development of this minority group is often ignored. There are no relevant literature reports on male nursing teachers in the field of nursing education in mainland China, which belongs to the research field blank. This study paid early attention to the professional environment of Chinese male nursing teachers, explored their development status and needs, put forward career development opinions based on their true attitudes, provided reference opinions for educational managers, and promoted the stability and ability of the nursing teacher group. It can also provide reference for the career development of male nursing teachers.

Currently, the number of male nursing teachers in China is rising rapidly. They can diversify the gender of the nursing education group and promote the stability of the nursing teacher team. Attract more men to choose nursing majors and improve social recognition of male nursing workers. The gender advantages of male nursing teachers in critical care, operating room operations, and first aid can also be used to improve students' professional judgment and operational abilities. Therefore, men are indispensable in the nursing teacher team. They play an important role and contribute to Chinese nursing education.

This study explored the feelings of MNT in universities through semi-structured in-depth interviews and five themes: realistic difficulties of the profession, positive feelings about the profession, sources of occupational stress, methods of coping with occupational stress, and career development expectations. These findings suggest that the construction of nursing faculties in universities needs to be further improved, the gender ratios and perceptions of nursing teachers need to be improved, and humanistic care needs to be strengthened. These changes will be conducive to further promoting the sustainable development of the nursing profession and the rationalization of the faculty in China and advancing the development of nursing education and the internationalization of the faculty in China.

### Limitation and recommendation

The findings of this study truly reflect the in-depth working experience of MNT in universities and can provide a reference for education managers to formulate policies and stabilize teaching teams. This study had some limitations. First, it adopts the strategy of maximum difference in sampling. However, due to geographical, time, and manpower constraints, the participants in this study were all MNT from various universities in Zhejiang Province, China. To a certain extent, this limitation affects the generalizability of the results. However, this study selected MNT from different regions of Zhejiang Province, including most universities with MNT. Therefore, the research area will be expanded in follow-up research to make the research objects more representative. Second, although this research reached saturation of the theme, it is a relative concept, and the results may change with the passage of time or the professional development of MNT. Therefore, we will continue to conduct in-depth research and focus on dynamic changes in this group.

Based on the relevant results of this study, the following recommendations were made: First, university administrators should pay attention to the gender ratio structure of nursing teachers, increase the number of MNT, strengthen vocational skills training, improve salaries and benefits, and establish a comprehensive career development program for this group to improve the career access and stability of MNT. Second, nursing education managers should establish personal development files of the entire career cycle for MNT, pay attention to dynamic changes in career psychology, provide psychological support channels, and improve humanistic care. Finally, male nursing faculty in higher education should seek to expand their voices in schools and society by establishing local male nursing faculty coalitions or groups at the organizational level to enhance the social presence and recognition of the group through academic conferences, policy contributions, social services, and public services.

## Data availability statement

The original contributions presented in the study are included in the article/supplementary material, further inquiries can be directed to the corresponding author.

## Ethics statement

The studies involving humans were approved by the Ethics Committee of the Affiliated Central Hospital of Shaoxing University. The studies were conducted in accordance with the local legislation and institutional requirements. The participants provided their written informed consent to participate in this study. Written informed consent was obtained from the individual(s) for the publication of any potentially identifiable images or data included in this article.

## Author contributions

ZH: Conceptualization, Data curation, Investigation, Project administration, Writing—original draft, Writing—review & editing. JY: Investigation, Project administration, Writing—original draft. MD: Project administration, Supervision, Writing—review & editing. XZ: Data curation, Investigation, Methodology, Project administration, Supervision, Validation, Writing—review & editing.
